# Digital Interventions to Enhance Inpatient Physical Activity in Forensic Mental Health Units: A Literature Review

**DOI:** 10.1192/j.eurpsy.2026.11289

**Published:** 2026-07-31

**Authors:** D. P. Calilung

**Affiliations:** 1 Forensic Psychiatry, Avon & Wiltshire Mental Health Partnership NHS Trust, Bristol; 2 Royal College of Psychiatrists, London, United Kingdom

## Abstract

**Introduction:**

There is growing research on digital interventions for physical health initiatives such as systematic reviews of mobile applications improving physical fitness in children (Wang et al., 2024) and digital exercise programmes on muscle function in older adults (de la Valle et al., 2025). Specialised group interventions also exist, as with the UK’s “Kidney BEAM” trial (Greenwood et al., 2024). These advances prompted the author to conduct a review on the specialised cohort of Forensic Psychiatry inpatients who face additional barriers to conventional physical activity due to restrictive secure environments, prolonged hospital stays, and antipsychotic-induced weight gain and metabolic effects.

**Objectives:**

To synthesise current evidence on the feasibility and preliminary outcomes of digital interventions on physical activity of inpatients at forensic mental health hospitals through a literature review.

**Methods:**

A database search (PubMed, PsycINFO, CINAHL, 2005–2025) was conducted and limited to publications in English. Data was extracted and organised using the literature matrix model by the University of Sheffield.

**Results:**

Two studies were identified: (1) virtual cycling (VC) in a medium-secure unit in Denmark (Kramer et al., 2025) and (2) exergaming via Wii-fit at a forensic hospital in Australia (Bacon et al., 2012). The VC qualitative study involved 5 male patients who reported increased levels of: exercise motivation, sociability with physiotherapists, patient empowerment, and reduced boredom. The Wii-fit study used mixed methods with 1 male and 1 female patient in twice-weekly sessions over 8 weeks. Both demonstrated increased time spent in moderate-intensity activity and modest weight loss i.e., 61 and 114 minutes, 3.4kg and 1kg, respectively, alongside positive feedback towards the sessions as “fun, motivating.”

A proposed study in Australia by Kaye et al. (2022) planned a non-randomised two-armed pilot study at a high-secure unit evaluating exergaming via Xbox Kinect over 12 weeks. It aimed to measure feasibility of recruitment and adherence with identified secondary outcomes of increased physical fitness, longer activity duration, and positive effects in metabolic markers. The study was halted due to COVID-19 restrictions and has not resumed to date.

**Conclusions:**

The literature review yielded extremely limited evidence. Findings suggest potential benefits of such interventions on physical health needs of the inpatients. However, methodological constraints of small samples, single-site designs, constrained access to technology, and added approval under legal frameworks, remain a challenge to pursue further research. This review highlights a call for wider studies re-examining health policies on the forensic population, and exploring collaborations between mental health and criminal justice systems that would improve equity of access to digital health initiatives.

**Disclosure of Interest:**

None Declared

